# Lung Adenocarcinoma Presenting as Malignant Pericardial Effusion/Tamponade

**DOI:** 10.7759/cureus.13762

**Published:** 2021-03-08

**Authors:** Lalitha Padmanabha Vemireddy, Nikita Jain, Ammar Aqeel, Hafiz Muhammad Jeelani, Maryna Shayuk

**Affiliations:** 1 Internal Medicine, Chicago Medical School Internal Medicine Residency Program at Northwestern McHenry Hospital, McHenry, USA

**Keywords:** malignant pericardial effusion, lung adenocarcinoma, tamponade

## Abstract

Lung cancers are the most common primary tumors that involve the pericardium with a prevalence of up to 50%. Usually, pericardial involvement goes undetected with almost 10%-12% found among all cancer related autopsies. Rarely pericardial effusions can be the initial site of metastasis and initial manifestation of a primary tumor. In our case, we report a 57-year-old female presenting with cardiac tamponade and subsequent testing was done which revealed lung adenocarcinoma. Malignant pericardial effusions are often silent, but certain times can present with symptoms of shortness of breath, chest pain, cough, arrhythmias, and rarely as pericardial tamponade. A high index of suspicion is required when a patient presents with tamponade to diagnose malignancy. Emergent pericardiocentesis may be warranted depending on the clinical presentation but quite often, patients tend to have a poor prognosis despite therapy given the extent of disease.

## Introduction

Malignant pericardial effusion is seen more frequently with non-cardiac malignancies than primary cardiac tumors and is usually detected post mortem, with a prevalence of 10%-12% among all cancer related autopsies [1,2]. Although rare, it can be the initial manifestation of a primary malignancy and can sometimes present as the initial site of metastasis [1]. Lung cancer is the most common cause of pericardial involvement and studies have shown a prevalence of about 33% to 50% among all malignant pericardial effusions. Breast cancer, with a known prevalence of 18% per Strobbe et al., is the second most common cause of malignant pericardial effusion [3]. Other common tumors involved are mediastinal lymphoma, melanoma, mesothelioma, renal cell carcinoma [1,3]. Ben-Horin et al. reported an incidence of 33% (58 out of 173 patients with malignant pericardial effusions), and 22% (13 out of 59) of these patients presented with symptoms of pericardial effusion as the initial manifestation of primary malignancy [4]. Here, in this report, we present a rare case of pericardial effusion with tamponade as the initial manifestation of lung adenocarcinoma.

## Case presentation

**Figure 1:**
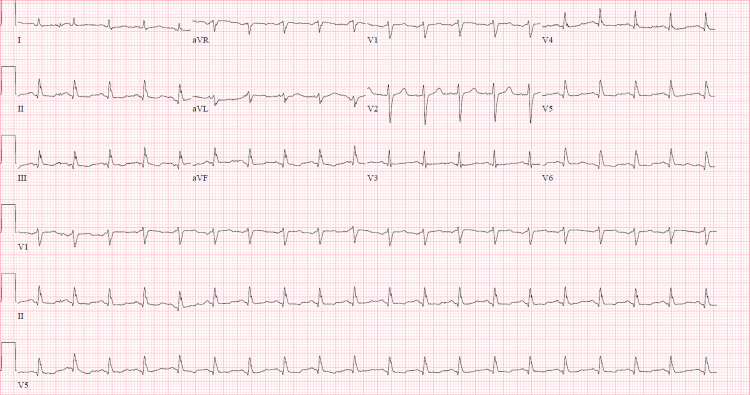
Electrocardiogram demonstrating sinus tachycardia with nonspecific ST-T segment changes.

We present a 57-year-old female with a history of hypertension who presented with worsening shortness of breath and chest pain for two days. Social history revealed that the patient was a never smoker. A review of systems was unremarkable except for bilateral leg pain with swelling. On physical examination, the patient was afebrile, tachycardic (heart rate of 127/min), blood pressure of 164/107 mmHg, with distant heart sounds, and bilateral (b/l) calf tenderness. Initial laboratory results showed a leukocytosis of 11.3 k/μL, brain natriuretic peptide at 45.5 pg/ml, troponin <0.04 ng/ml with the rest of the lab values being normal. Electrocardiogram on presentation showed sinus tachycardia without any ST or T wave changes or electrical alternans (Figure 1).

**Figure 2:**
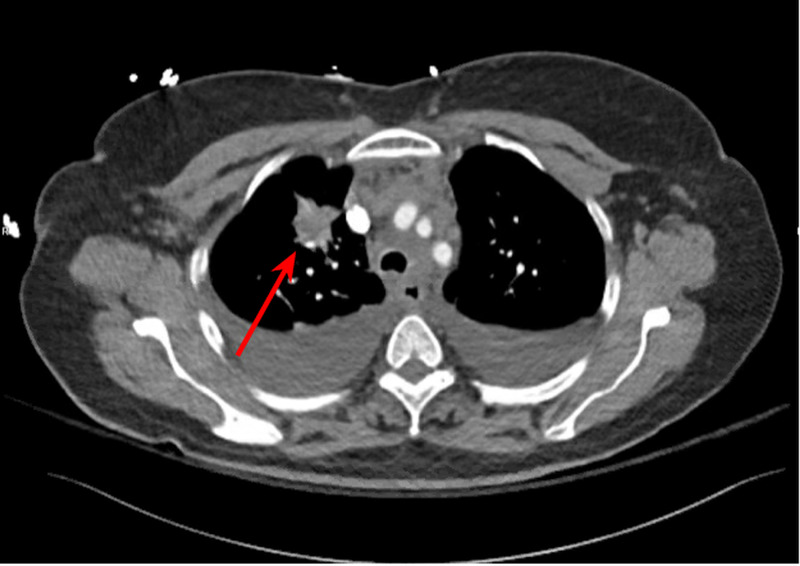
CT angiogram of the chest showing a spiculated mass in the right upper lobe (arrow).

Imaging in the emergency department included a computed tomography (CT) angiography of the chest which showed several non-occlusive pulmonary emboli in lateral basal segmental/sub-segmental and anterior basal segmental artery of left lower lobe, apical segmental artery of right upper lobe, medial segmental artery, proximal right middle lobar artery of the right middle lobe, along with a spiculated mass within the right upper lobe (Figure 2).

**Figure 3:**
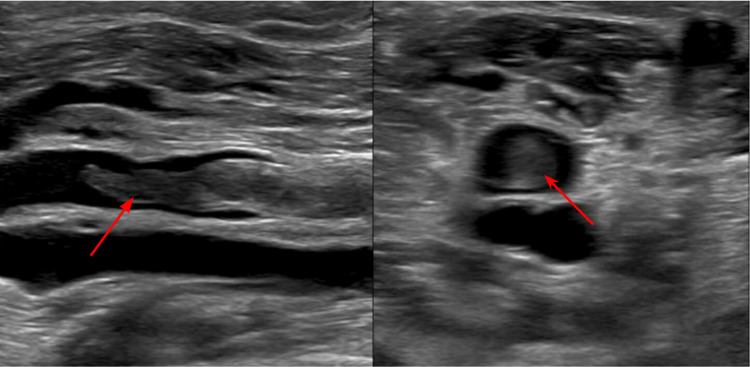
Ultrasound of the lower extremity showing thrombus in the right distal popliteal vein (arrow) in different views of ultrasound.

An ultrasound of b/l lower extremities showed extensive b/l deep vein thrombosis (Figure 3).

**Figure 4:**
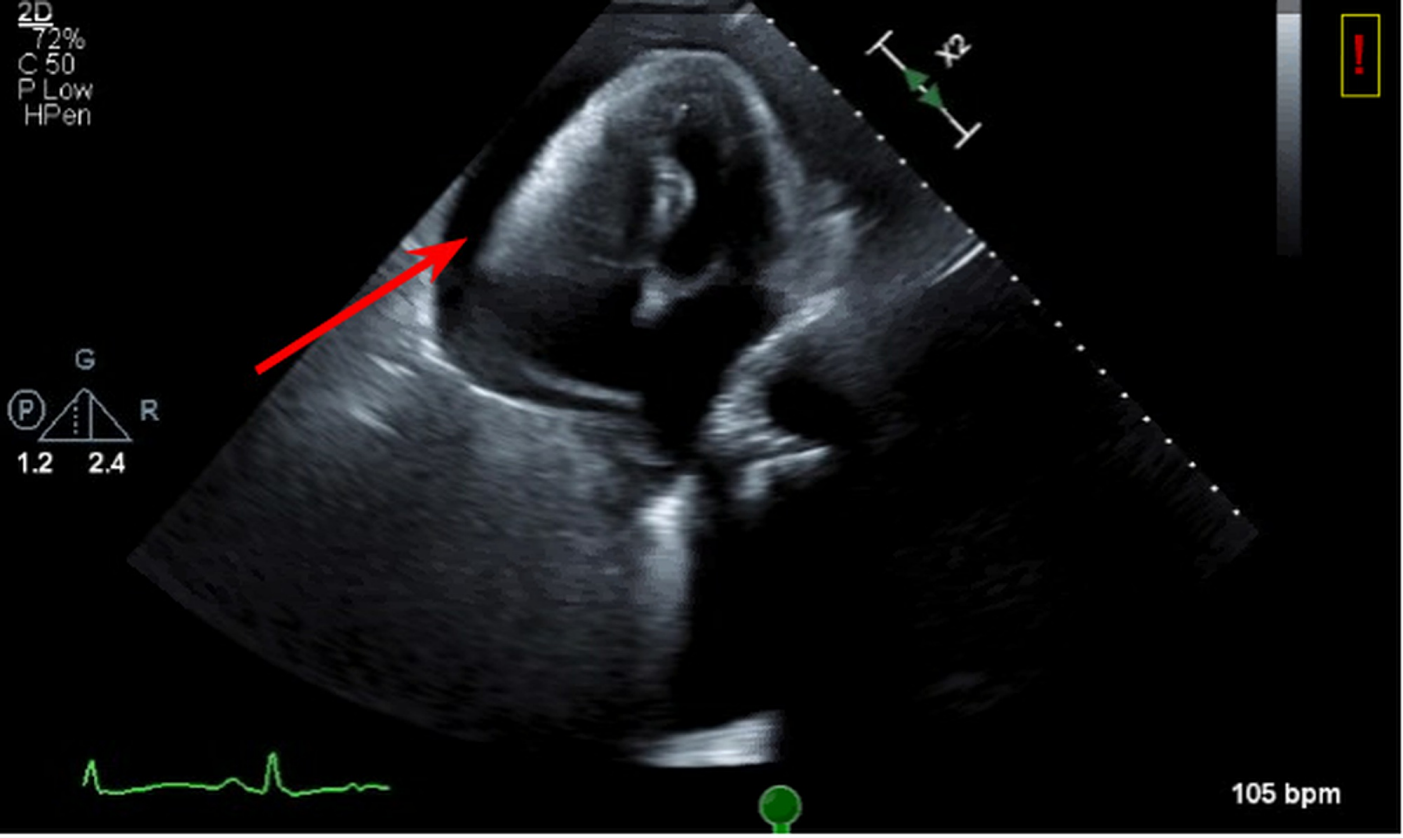
2D echocardiogram on apical view showing fluid around the right atrium and ventricle with a completely collapsed right ventricle (arrow).

Echocardiogram was done to rule out right heart strain given the large burden of pulmonary embolism. It showed a large pericardial effusion with collapse of both right atrium and right ventricle (Figure 4).

**Figure 5:**
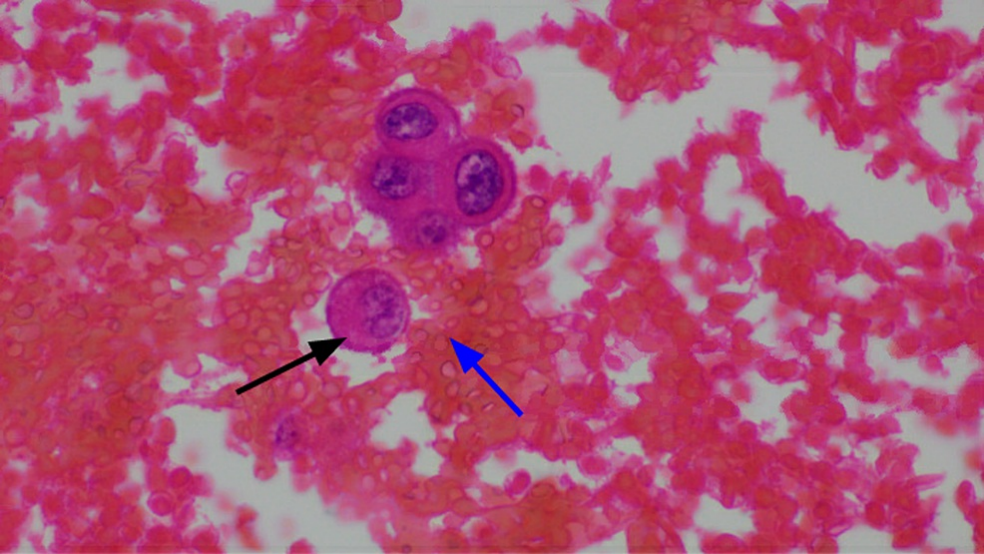
Hematoxylin and eosin staining showing the malignant adenocarcinoma cell (black arrow) with background of hemorrhagic effusion (blue arrow).

**Figure 6:**
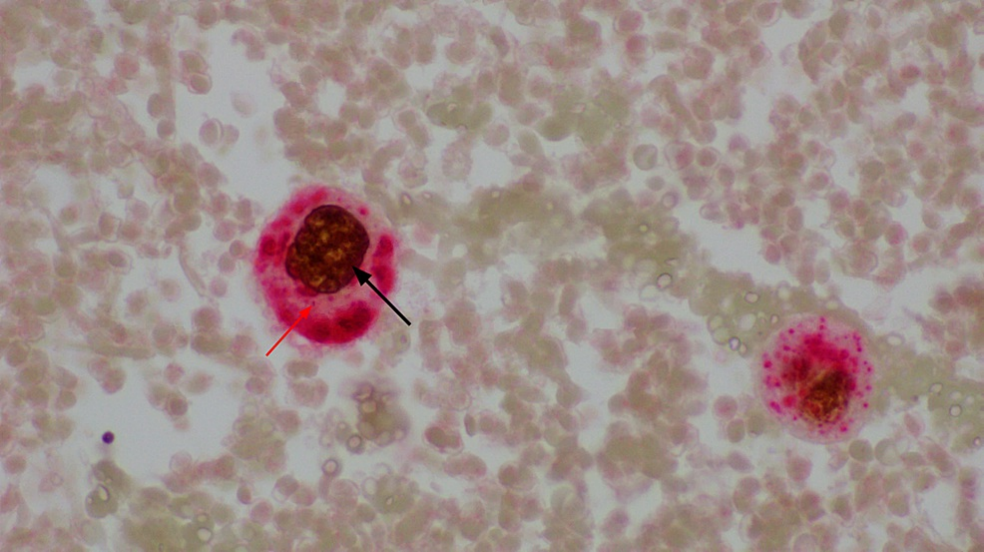
IHC showing TTF-1 (black arrow) staining of the nucleus of malignant adenocarcinoma cell of the lung with napsin-A (red arrow) staining the cytoplasm. TTF-1: thyroid transcription factor-1; IHC: immunohistochemistry.

An emergent pericardiocentesis was pursued due to persistent tachycardia, distant heart sounds, and evidence of tamponade with collapse of both right atrium and ventricle. About 400 ml of hemorrhagic pericardial fluid was removed. Analysis of the fluid revealed malignant epithelial cells consistent with metastatic lung adenocarcinoma. Pathology showed strong positivity for cytokeratin 8-18, thyroid transcription factor-1 (TTF-1), napsin A, negative for anaplastic lymphoma kinase (ALK)/epidermal growth factor receptor (EGFR), and pending programmed death-ligand 1 (PD-L1) status (Figures 5-6).

**Figure 7:**
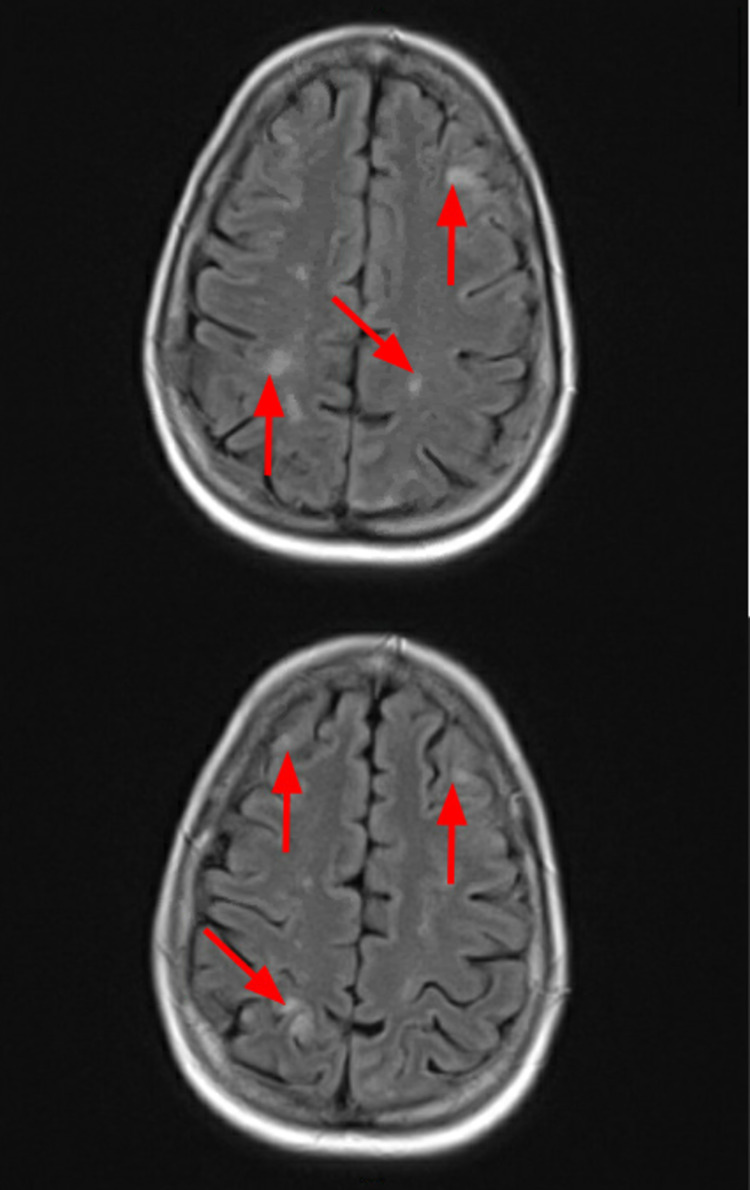
MRI brain images showing multiple lesions (arrows) in the cortex of the brain.

After pericardiocentesis, the rest of the hospital course was uneventful and the patient was discharged home. Further workup upon outpatient follow up with MRI brain showed multiple 2-5 mm enhancing lesions in the posterior fossa and supratentorial brain, consistent with metastasis (Figure 7).

Patient was initiated on carboplatin, pemetrexed, and pembrolizumab for further treatment of adenocarcinoma of the lung.

## Discussion

About 90% of cases of secondary cardiac tumors causing malignant pericardial effusion are clinically silent and thus, mostly found on necropsy [1]. In rare instances, patients present with symptoms such as shortness of breath, chest pain, cough, or palpitations [2,5]. In extreme cases, arrhythmias may be the initial complaint/presentation which should warrant consideration of myo-epicardial/pericardial disease involving the conduction system [2].

Diagnosis requires a high index of suspicion as presentation can be variable. Physical examination findings like elevated jugular venous pressure (JVP), hypotension, distant heart sounds, and pulsus paradoxus can be present and are suggestive of cardiac tamponade, but may be absent in smaller effusions [6]. Other nonspecific findings include cardiomegaly on chest radiograph, and electrical alternans or low amplitude complexes on an EKG. Echocardiography remains the initial imaging for diagnosis, showing the effusion with or without tamponade. Cardiac CT and MRI have also been used which provide a detailed view and help in differentiating the tumor from myocardium [2,7]. Frank tamponade and bloody effusions are more common in malignant pericardial effusion than benign causes of effusion [5,8]. Cytology for malignant pericardial effusion had a 51% sensitivity in a study done by Ben-Horin et al. and can range from anywhere between 66.7% to 92% based on other studies [3,4].

Treatment varies based on the size of effusion and clinical presentation. In cases of tamponade with hemodynamic instability, emergent pericardiocentesis is warranted as this provides immediate relief [7,9]. In stable patients, non-emergent percutaneous or open pericardiotomy may be considered [10]. Although complication rates are lower with prolonged pericardiocentesis, the diagnostic yield and recurrence rates are similar to pericardiotomy [10]. Labbe et al. reported 2.5 months of survival rate both with pericardiocentesis and pericardiotomy [5]. Malignant pericardial effusion has a very poor prognosis especially in patients with a known history of cancer prior to presentation [3,7,11]; this is likely related to the extent of the disease.

## Conclusions

Cardiac tamponade can arise secondarily from a wide range of etiologies and have varying presentations depending on the cause. Generally known to be a slowly developing and clinically silent disease process, malignant pericardial effusions can seldom present with hemodynamic instability and be the initial manifestation of an underlying malignancy. In this review, we present a rare case of metastatic lung adenocarcinoma manifesting as cardiac tamponade. If this presentation is not recognized and managed emergently, the results might be fatal, thus, it is paramount for clinicians to be aware of such atypical presentations of thoracic malignancies.
